# Contagious statistical distributions: *k*-connections and applications in infectious disease environments

**DOI:** 10.1371/journal.pone.0268810

**Published:** 2022-05-27

**Authors:** Victoriano García–García, María Martel–Escobar, Francisco–José Vázquez–Polo

**Affiliations:** 1 Department of Statistics and O.R., Faculty of Economics, University of Cádiz, Cádiz, Spain; 2 Department of Quantitative Methods and TiDES Institute, Faculty of Economics, University of Las Palmas de Gran Canaria, Las Palmas de Gran Canaria, Canary Islands, Spain; Utrecht University, NETHERLANDS

## Abstract

Contagious statistical distributions are a valuable resource for managing contagion by means of *k*–connected chains of distributions. Binomial, hypergeometric, Pólya, uniform distributions with the same values for all parameters except sample size *n* are known to be strongly associated. This paper describes how the relationship can be obtained via factorial moments, simplifying the process by including novel elements. We describe the properties of these distributions and provide examples of their real–world application, and then define a chain of *k*–connected distributions, which generalises the relationship among samples of any size for a given population and the Pólya urn model.

## Introduction

A large body of literature has been generated regarding mathematical models of epidemiology. These models usually consider the population under study to be clustered as follows: persons born with passive immunity (denoted by M), those without passive immunity and hence susceptible (S), those who are infected but not infectious (E), those who are capable of transmitting the infection, and hence infectious (I), and those who have a permanent infection–acquired immunity, and hence recovered (R). Different epidemiology models are classified according to which of these clusters are considered: MSEIR, SEIR, SIR, etc.

Among the main parameters included in these models are the basic reproduction number of an epidemic, *R*_0_ (that is, the expected number of secondary cases produced by a primary case during their infectious period, within a completely susceptible population), and the degree of herd immunity (the fraction of immune individuals within the population beyond which the epidemic can no longer grow). A brief historical summary of mathematical models in epidemiology can be found in Hethcote [1].

Knowledge and understanding in this area have advanced rapidly, and many empirical studies have improved upon the classical models by including significant features such as the effects of heterogeneity and correlations, household effects, network–driven contagion and mobility models. Sun et al. [2], in a study based on Chinese data, considered the role of transmission heterogeneities, which are driven by demography, behaviour and interventions. Kawagoe et al. [3]examined the question of infectious disease dynamics in heterogeneous populations and the role played by “superspreaders”. Aleta et al. [4] studied the effects of testing, quarantine and contact tracing, and Huber et al. [5] proposed a tracing strategy to optimise the cost/effect balance. Chang et al. [6] successfully developed a SEIR model which used mobile phone geolocation data.

Most of the mathematical models proposed require certain assumptions about the dynamics of infectious disease. For instance, a common and sometimes unrealistic assumption is that there is the same probability of any infectious individual infecting any susceptible one, a relation that is termed homogeneity. Britton et al. [7] showed that the contrary situation, that of population heterogeneity, can have a considerable impact on disease–induced immunity because the proportion of infected individuals in groups with higher contact rates is greater than that in groups with lower contact rates. Hébert–Dufresne et al. [8] showed, using random network theory to predict the size of an epidemic, that without data on the heterogeneity in secondary infections (which are needed to estimate its cumulative distribution function) the size of the outbreak remains highly uncertain.

Seeking to avoid the above assumption, various improvements to the models have been suggested. For instance, Neipel et al. [9] generalised the SIR model taking into account generic effects of heterogeneity on the population’s degree of susceptibility to infection. Introducing a new parameter, that of a power–law exponent of the susceptibility distribution at small susceptibilities, Neipel et al. showed that the class of gamma distributions acts as an attractor of the dynamics, making it possible to identify generic effects of population heterogeneity.

Another common assumption which may be unrealistic is the “law of large numbers” (LLN), meaning that the population size is large enough to accurately describe random dynamics with asymptotic elements, such as limit probability distributions. However, many situations of infectious disease spread originate within a closed environment (school classrooms, for instance) with a population size, where the LLN assumption does not hold. In this circumstance, attempting to forecast the behaviour of infection dynamics by means of the classical models would be quite misleading. Brooks et al. [10] developed a stochastic transmission model of infection spread in university campuses, based on realistic mixing patterns, and evaluated various infection mitigation strategies. Mayberry et al. [11] presented dynamic random graph techniques for modelling small population outbreaks, allowing different interaction rates among students. These authors analysed Monte Carlo simulations, assuming a beta negative binomial distribution, to determine the effects of different transmission rates and of diverse vaccination strategies on the dynamics of a hypothetical outbreak of influenza. With respect to the COVID–19 pandemic, several guidelines on appropriate antigen–testing strategies have been developed. For instance, the National Academies of Sciences, Engineering, and Medicine [12] provided a general guide for colleges and universities in the USA, and Nixon et al. [13] described how the University of Bristol (UK) developed CONQUEST, a tool to record and analyse data on COVID–19.

In this paper, we consider contagious statistical distributions and theoretical tools which could be applied to certain scenarios of infection, such as a closed environment with several rooms. In addition, we present techniques showing how complementary information on the statistical behaviour of infectious disease spread can be obtained.

Contagious statistical distributions are valuable toolboxes relating to the epidemiology of communicable diseases. These resources enhance our understanding of the presence of contagions in, for example, a confined space. In a more general scenario, assume a system with *n* components. Each component could have a different workload and hence a different probability of failing. The variable considered is the number of failure events. Now assume a different number of system components and a different workload for each component; nevertheless, the overall proportion of failing components remains unaltered. The question then arises: when *X*^(*n*)^ is the number of fails when the system contains *n* components, what relationship exists (if any) among the probability distributions of *X*^(*n*)^ for *n* = 1, 2, …?

Such a scenario is most commonly modelled using a classical binomial distribution, in which each component has the same probability of failing. However, this means there may be a high variance, and therefore a large statistical error in the estimates obtained. A second concern is the implicit assumption that the binomial probability mass function (pmf) seems to resemble a Gaussian curve (as the *n* increases), producing a certain symmetry, unimodality, etc. This assumption is not always valid. Finally, independence cannot always be assumed.

The Pólya urn (contagious) model described by Eggenberger and Pólya [14] models the above situation by considering an urn which initially contains *W* white balls (cases) and *R* red ones (others). One ball is sampled at random and returned to the urn with *c* additional balls of the same colour. After this procedure has been applied to *n* samples, the variable, *X*^(*n*)^, which counts the number of white balls sampled is said to be Pólya distributed, and is denoted by *X*^(*n*)^ ∼ P(*W*, *R*, *c*, *n*). Its pmf, i.e. the probability that, after *n* draws, *w* white balls (representing cases of infection) and *n* − *w* = *r* red balls (representing individuals free of infection) have been drawn, is given by

$$\begin{array}{ccc} \;\text{Pr}\;(X^{\left(n\right)}=w|W,R,c,n) & = & \frac{n!}{w!r!}\frac{p(p+\delta )\cdots [p+(w-1)\delta ]}{1(1+\delta )\cdots [1+(w-1)\delta ]}\\ & & \times \frac{q(q+\delta )\cdots [q+(r-1)\delta ]}{\left(1+w\delta )[1+(w+1)\delta ]\cdots [1+(n-1)\delta \right]} \end{array}$$ (1)

where *p* = *W*/(*W* + *R*), *q* = 1 − *p*, and *δ* = *c*/(*W* + *R*), subject to the following feasibility conditions:

1. *W* + *R* − 1 ≥ (1 − *n*)*c*, to have a feasible set of parameters, and
2. min{*W* − 1, *R* − 1} ≥ (1 − *n*)*c*, to have a distribution in which the rank is complete.

Particular cases are:

1. Let *np* = *h*, *nδ* = *d*, and *n* → ∞, with *h* and *d* remaining finite, then (1) has the limiting form
  $$\begin{array}{c} \frac{h(h+d)(h+2d)\cdots [h+(w-1)d]}{w!{\left(1+d\right)}^{(h/d)+w}}, \end{array}$$
  which corresponds to a negative binomial distribution.
2. For *c* = 0, then (1) reduces to a classical binomial model, Bin(*n*, *p*).
3. If negative values are allowed for *c*, then for *c* = −1, P(*W*, *R*, −1, *n*) reduces to the hypergeometric model, H(*W* + *R*, *W*, *n*) of sampling without replacement.

Recent studies of the Pólya urn models include in Kotz et al. [15], Mahmoud [16], Chen and Wei [17] and Chen and Kuba [18].

Ollero and Ramos [19] showed that the Pólya distributions (allowing any feasible integer value for *c*) are equivalent to Poisson–Binomial models. The Poisson–Binomial model describes the number of successes, *X*^(*n*)^, in *n* Bernoulli independent trials, each of which has the probability of success *p*_*i*_, *i* = 1, …, *n*. The model is denoted by *X*^(*n*)^ ∼ PB(**p**), where **p** = (*p*_1_, …, *p*_*n*_), and its pmf is given by

$$\begin{array}{c} \;\text{Pr}\;(X^{\left(n\right)}=k)=(\prod_{i=1}^{n}\left(1-p_{i}\right))(\sum_{i_{1}<\ldots <i_{k}}\text{logit}\left(p_{i_{1}}\right)\cdot \ldots \cdot \text{logit}\left(p_{i_{k}}\right)), \end{array}$$ (2)

where logit(*p*_*i*_) = *p*_*i*_/(1 − *p*_*i*_), for *i* = 1, …, *n*, and the summation is over all possible combinations of different *i*_1_, …, *i*_*k*_ from {1, …, *n*}. Clearly, the mean of the random variable *X*^(*n*)^ is given by

$$\begin{array}{c} E(X^{\left(n\right)})=p=\sum_{i=1}^{n}p_{i}. \end{array}$$

This main result from [19] is quite surprising, meaning that the number of successes in *n* dependent Bernoulli trials can be described as the number of successes in independent ones.

The Poisson–Binomial distribution has had relatively little research attention in recent years, mainly due to the absence of assumptions regarding its parameters: the Poisson binomial family contains quite different distributions, with quite different properties, and *n* parameters are required to model a random variable which can take *n* + 1 values. Among the few more or less recent papers on these distributions, theoretical results have been reported by Schlemm [20] and a goodness of fit test was proposed by Acharya and Daskalakis [21]. In addition, some work on approximation, by different methods, has been done by Neammanee [22, 23] and Barbour [24], Skipper [25], Butler and Stephens [26] and Novak [27]. Studies related to the computation of probabilities include Hong [28] and Barrett and Gray [29]. Analyses in which the model has proven useful are described in Chen and Liu [30], Tejada and den Dekker [31] and Rosenman and Viswanathan [32]. An excellent review of the most recent progress related with the Poisson–Binomial distribution is Tang and Tang [33].

Let us assume that not only Poisson–Binomial models but any finite distribution might best fit the data. If the cdf of each *X*^(*n*)^ is denoted by *F*^(*n*)^ then we wish to find chains of distributions of the form {*F*^(*n*)^: *n* = 0, …, *M*}, where *M* could be infinity or an integer upper bound to the chain, and where all these distributions share certain regularity conditions. However, these conditions cannot be defined in a simple way.

A chain of finite distributions has a relationship called *k*–connection, meaning there exists a strong relationship among the respective factorial moments, which can be viewed as a regular pattern of behaviour within a contagious environment. This, in turn, implies the existence of proportionality in the expected means, variances, etc., thus providing us with an instrument to manage the behaviour of the number of infections taking place in an environment as its population increases.

By means of this relationship, a model for the number of successes in *n* = *n*_0_ trials can not only be described by a given distribution *F*^(*n*)^, but can also facilitate a chain of *k*–connected distributions for any other feasible sample size. When the relationship among the models within a chain is assumed as part of the model, it can be tested or estimated from samples of different sizes.

The aim of this paper is to describe and/or characterise families of discrete distributions parametrised by a sample size. These distributions are used to model contagion via a relationship that we term *k*−connectedness. We show that this relationship can be presented in a natural way, among many well–known families of discrete distributions, such as the chains {Bin(*n*, *p*): *n* ≥ 0}, {P(*W*, *R*, −1, *n*): *n* = 0, …, *M*}.

What is this relationship useful for? Theorem 1 shows that chains of connected distributions are feasible statistical models for estimating the proportion of infected individuals in a population, given samples of varying sizes from this population. In other words, sample observations would commonly be used, jointly, with different sample sizes to estimate a unique or common value for the probability of success, *p*. Thus, data from different distributions within a chain of connected distributions can be jointly used for inference. This powerful possibility is proven to be feasible within any given chain of *k*−connected distributions.

The rest of this paper is organised as follows. Next, we present the following theoretical elements considered, and describe their main properties: the connecting function of a finite random variable or distribution; the *k*–connection relationship; the chains of connected distributions; and the chain–generating sequence. In addition, we provide a triangular table to represent a chain generating sequence. Finally, some subsets of well–known families of finite distributions are shown to be chains of connected distributions. In the Estimation section, we then illustrate a practical application of these elements, with a real–world example of their use, showing that samples from different distributions belonging to the same chain of *k*–connected ones can be used jointly for estimation. A simulation study is also performed to rule out the possibility of errors in the estimation process. Finally, we summarise the main conclusions drawn.

## Chain of distributions

In this section, we define and study some auxiliary elements to simplify the definition of a chain of *k*–connected distributions. Instead of addressing this relationship by means of factorial moments, we do so using a characteristic function of the distributions, termed the connecting function. To facilitate the detection and management of a chain of *k*–connected distributions, we also define the chain generating sequence, i.e. the sequence of real numbers that characterises a given chain of *k*–connected distributions. Some classical (but previously unknown) chains and their generating sequences are also shown.

**Definition 1** *X*^(*n*)^ *be a random variable with support in the integer interval* [0, *n*]. *The function*

$$\begin{array}{c} C(z)=E[z^{n-X^{\left(n\right)}}{\left(z-1\right)}^{X^{\left(n\right)}}],\;\forall z>0, \end{array}$$ (3)

*is then termed the connecting function of X*^(*n*)^.

Expression (3) can be rewritten in terms of the probability generating function (pgf) $G\left(z\right)=E(z^{X^{\left(n\right)}}),$

$$\begin{array}{c} C(z)=z^{n}E[{\left(\frac{z-1}{z}\right)}^{X^{\left(n\right)}}]=z^{n}G(\frac{z-1}{z}),\;\forall z>0. \end{array}$$ (4)

**Example 1** *For a binomial random variable X*^(*n*)^ ∼ *Bin*(*n*, *p*) *the connecting function is given by*

C(z)=E[zn−X(n)(z−1)X(n)]=∑x=0n(z−1)xzn−x(nx)px(1−p)n−x=∑x=0n(nx)((z−1)p)x(z(1−p))n−x=(z−p)n.

**Proposition 1** *The connecting function of a Poisson–Binomial distributed variable*, *X*^(*n*)^ ∼ *PB*(*p*_1_, …, *p*_*n*_) *is given by*

$$\begin{array}{c} C(z)=\prod_{i=1}^{n}(z-p_{i}). \end{array}$$

*Proof*. Given that the well–known Poisson–Binomial probability generating function can be expressed as

$$\begin{array}{c} G(z)=\prod_{i=1}^{n}(1-p_{i}+p_{i}z), \end{array}$$

the proof follows immediately from (4).

**Corollary 1** *The connecting function of a random variable X*^(*n*)^ *with support on the integer interval* [0, *n*] *is a polynomial with real roots iff X*^(*n*)^ *is a Poisson–Binomial distributed variable*.

*Proof*. To prove this, it only has to be noticed that any real root of C(z) is inside the real interval [0, 1].

The connecting function is no more than a particular probability generating function. Nevertheless, it is a useful means of presenting the natural concept of chain of connected distributions, which to our knowledge has not been addressed before. In this understanding, we first introduce the concept of *k*−connection and then go on to prove that it is the common internal relationship of certain particular sets of discrete probability distributions.

**Definition 2** *Let X*^(*n*)^ *and X*^(*n*+*k*)^ *be random variables with respective connecting functions* $C_{n}(z)$ *and* $C_{n+k}(z)$ . *Both variables and their respective distributions are said to be k*–*connected if*

$$\begin{array}{c} \frac{d^{k}}{dz^{k}}C_{n+k}(z)=\frac{(n+k)!}{n!}C_{n}(z). \end{array}$$

When a pair of random variables, *X*^(*n*)^ and *X*^(*n*+1)^, are 1–connected, they are said to be connected.

For instance, in the binomial distributions Bin(*n*, *p*) and Bin(*n* + 1, *p*), we have that $C_{n+1}\left(z\right)={\left(z-p\right)}^{n+1},$ and so Bin(*n*, *p*) and Bin(*n* + 1, *p*) are 1–connected. Analogously, Bin(*n*, *p*) and Bin(*n* + 2, *p*) are 2–connected distributions, and so on. The same outcomes are obtained in most classical finite models, such as Pólya distributions and discrete uniform distributions.

In the following, we use the standard Pochhammer notation for the falling and rising factorials:

$$\begin{array}{ccc} {\left[a\right]}_{i} & = & a\cdot (a-1)\cdot \ldots \cdot (a-i+1),\\ {\left(a\right)}_{i} & = & a\cdot (a+1)\cdot \ldots \cdot (a+i-1),\;{\left(a\right)}_{0}=1, \end{array}$$

and ${\left[\mu \right]}_{i}(X)=E[X(X-1)\cdots (X-i+1)]$.

The following properties are straightforwardly proven.

**Proposition 2** *Let X*^(*n*)^ *and X*^(*n*+1)^ *be connected random variables with respective connecting functions* $C_{n}\left(z\right),$ *and* $C_{n+1}\left(z\right).$ *Let h* ∈ {*n*, *n* + 1}. *Then*:

1. $C_{h}\left(0\right)={\left(-1\right)}^{h}\;\text{Pr}\;(X^{\left(h\right)}=h).$
2. $C_{h}\left(1\right)=\;\text{Pr}\;(X^{\left(h\right)}=0).$
3. *The connecting function can also be written as*
  $$\begin{array}{c} C_{h}\left(z\right)=\sum_{i=0}^{h}{\left(-1\right)}^{i}\frac{{\left[\mu \right]}_{i}\left(X^{\left(h\right)}\right)}{i!}z^{h-i}. \end{array}$$
4. *For i* = 0, …, *n* − 1, *this verifies*
  - (*n* + 1 − *i*)[*μ*]_*i*_ (*X*^(*n*+1)^) = (*n* + 1)[*μ*]_*i*_ (*X*^(*n*)^).
  - (*n* + 1) Pr (*X*^(*n*)^ = *i*) = (*n* + 1 − *i*) Pr (*X*^(*n*+1)^ = *i*)+ (*i* + 1) Pr (*X*^(*n*+1)^ = *i* + 1).
5. $Var(X^{\left(n+1\right)})=\frac{{\left(n+1\right)}^{2}}{n^{2}}Var(X^{\left(n\right)})+\frac{n+1}{n^{2}\left(n-1\right)}(E({X^{\left(n\right)}}^{2})-nE(X^{\left(n\right)}))$.
6. $C_{n+1}(z)={\left(-1\right)}^{n+1}\;\text{Pr}\;(X^{\left(n+1\right)}=n+1)+(n+1)\int_{0}^{z}C_{n}(t)\;dt.$

*Proof*. Parts 1 and 2 are straightforward from (3). To prove 3, denote by *f*_*h*,*i*_ = Pr(*X*^(*h*)^ = *i*), for *i* = 0, …, *h*. Then, expanding (*z* − 1)^*i*^ in (3) we have

Ch(z)=∑i=0h∑j=0i(−1)jfh,i(ij)zh−j=∑j=0n(−1)j(∑i=jh[i]jj!fh,izh−j),

Part b in 4 follows from

$$\begin{array}{c} C_{h}\left(z\right)=\sum_{i=0}^{h}f_{h,i}z^{h-i}{\left(z-1\right)}^{i}, \end{array}$$

and taking into account that $\frac{d}{dz}C_{n+1}\left(z\right)=\left(n+1\right)C_{n}\left(z\right).$ The remaining properties are obtained immediately.

It is obvious that if *X*^(*n*)^, *X*^(*n*+*k*)^ are *k*–connected and *X*^(*n*+*k*)^, *X*^(*n*+*k*+*h*)^ are *h*–connected, then *X*^(*n*)^, *X*^(*n*+*k*+*h*)^ are *k* + *h*–connected. Accordingly, this can be considered a sequence of consecutively connected variables, meaning that any pair of them are *k*–connected.

Notice that item 3 in Proposition 2 gives a recurrence relationship among the distributions. This relationship is verified by the well–known subfamilies of discrete distributions which are applied to *n*–sampling from a given population.

**Definition 3** *A set of random variables X*^(0)^, *X*^(1)^, … *such that any pair of them are k*–*connected for the appropriate k is said to be a chain of connected distributions*.

A chain of connected distributions can be finite or infinite, depending on its nature, and its first element is a degenerate random variable which takes a null value with full probability. In an example below, we demonstrate that binomial chains contain one distribution for each sample size. However, a chain of hypergeometric distributions only contains a finite number of ones, as the samples without replacement cannot be higher than the population. Given a discrete distribution *F*^(*n*)^ on {0, …, *n*}, it is easily proven that there exists a chain of connected distributions {*F*^(*k*)^: *k* = 0, …, *n*} which contains *F*^(*n*)^. The question to be solved is whether there exists an additional distribution *F*^(*n*+1)^ that would extend the chain.

Any chain of connected distributions is characterised by a sequence of real numbers, such that the chain can be extended if this is possible, and if not, this is apparent. These distributions are termed chain generating sequences. In this case, the finite difference operator of a sequence of numbers is denoted as

$$\begin{array}{ccc} \Delta^{0}a_{k} & = & a_{k}\\ \Delta^{j}a_{k} & = & \Delta^{j-1}a_{k+1}-\Delta^{j-1}a_{k},\;j=1,2,\ldots \end{array}$$ (5)

The following properties of this operator are evident:

**Lemma 1** *Given a sequence of real numbers A* = {*a*_*k*_: *k* = 0, …, *n*}, *then*

Δjak=∑i=0j(−1)i(ji)ak+j−i=∑i=0j(−1)j−i(ji)ak+i. (6)

**Definition 4** *Let A* = {*a*_*k*_: *k* = 0, …, *n*} *be a sequence of real numbers that verifies*:

1. *a*_0_ = 1
2. (−1)^*j*^ Δ^*j*^ *a*_*k*_ ≥ 0, for *k* + *j* ≤ *n*.

*Then, A is termed a chain generating sequence (cgs)*.

It can be easily proven that each element of a cgs lies within the real interval [0, 1], and that *a*_*k*_ ≥ *a*_*k* + 1_, for any *k* = 0, …, *n* − 1.

**Lemma 2** *Let X*^(*n*−1)^ *and X*^(*n*)^ *be random variables, and let*

$$\begin{array}{c} f_{n-1,i}=\;\text{Pr}\;(X^{\left(n-1\right)}=i),\;f_{n,j}=\;\text{Pr}\;(X^{\left(n\right)}=j), \end{array}$$

*for i* = 0, …, *n* − 1 *and j* = 0, …, *n*. *Then, X*^(*n*−1)^ *and X*^(*n*)^ *are connected iff it is verified that*:

$$\begin{array}{c} f_{n-1,i}=\frac{(n-i)f_{n,i}+(i+1)f_{n,i+1}}{n}, \end{array}$$

*for all i* = 0, …, *n* − 1.

*Proof*. Following part b in 4 from Proposition 2, we obtain one direction of the iff. To prove the opposite direction, and for *h* ∈ {*n* − 1, *n*}, the polynomial expression of $C_{h}\left(z\right)$ is given by

$$\begin{array}{c} C_{h}(z)=\sum_{i=0}^{h}f_{h,i}{\left(z-1\right)}^{i}z^{h-i}. \end{array}$$

Thus,

$$\begin{array}{c} \frac{dC_{n}(z)}{dz}=\sum_{i=0}^{n-1}((n-i)f_{n,i}+(i+1)f_{n,i+1}){\left(z-1\right)}^{i}z^{n-1-i}. \end{array}$$ (7)

After identifying the terms in the polynomial expression of $C_{n-1}$ we obtain that $\frac{dC_{n}\left(z\right)}{dz}=nC_{n-1}\left(z\right)$ is equivalent to verifying (7), and so the proof is complete.

**Theorem 1** *Let A* = {*a*_*k*_: *k* = 0, …, *N*} *be a cgs, where N could be infinite. Consider the set of vectors* **f**_*k*_ = (*f*_*k*,0_, …, *f*_*k*,*k*_), *where*

fk,i=(−1)k−i(ki)Δk−iai,i=0,…,k (8)

*Then, the set of random variables* {*X*^(*k*)^: *k* = 0, …, *N*} *such that*

$$\begin{array}{c} \;\text{Pr}\;(X^{\left(k\right)}=j)=f_{k,j},\;j=1,\ldots ,k, \end{array}$$

*is a chain of connected distributions, which we term the chain of connected distributions generated from A*.

*Proof*. Proceed recursively. For *k* = 1, *f*_0,0_ = *a*_0_ = 1, and *f*_1,0_ = Δ^1^*a*_0_ = 1−*a*_1_ ≥ 0, *f*_1,1_ = Δ^0^*a*_1_ = *a*_1_. Notice that *f*_0,0_ = (*f*_1,0_ + *f*_1,1_)/1, and apply Lemma 2. Now, if the result is true for *k* − 1, we search for a pmf **f**_*k*_ = (*f*_*k*,0_, …, *f*_*k*,*k*_) which is connected to **f**_*k*−1_ = (*f*_*k*−1,0_, …, *f*_*k*−1,*k*−1_). From Lemma 2, the following linear system must be solved:

$$\begin{array}{c} f_{k-1,i}=\frac{(k-i)f_{k,i}+(i+1)f_{k,i+1}}{k},\;i=0,\ldots ,k-1. \end{array}$$

Notice that condition $\sum_{i=0}^{k}f_{k,i}=1$ is redundant, and that the system has infinitely many solutions. A particular solution can be found by first taking *f*_*k*,*k*_ = (−1)^0^Δ^0^*a*_*k*_ = *a*_*k*_. After some straightforward, if tedious, calculus, the proof is complete.

Given any given finite distribution *F*^(*n*)^ within the integer interval [0, *n*] it is simple to obtain a chain that contains *F*^(*n*)^, and to determine whether another distribution *F*^(*n*+1)^ could be added to the chain. The necessary procedure, which somewhat resembles Pascal’s triangle, only requires the use of (8) and (5), as shown in the following example.

**Example 2** *Let X*^(2)^ *be a random variable with a pmf given by* $f_{2}=(f_{2,0},f_{2,1},f_{2,2})=(\frac{1}{6},\frac{2}{3},\frac{1}{6})$ , *and where*

$$\begin{array}{c} \;\text{Pr}\;(X^{\left(2\right)}=j)=f_{2,j}, \end{array}$$

*for j* = 0, 1, 2. *Then, from* (8) *we have*

$$\begin{array}{c} f_{2,0}=\frac{1}{6}=\Delta^{2}a_{0};\;f_{2,1}=\frac{2}{3}=-2\Delta^{1}a_{1};\;f_{2,2}=\frac{1}{6}=\Delta^{0}a_{2}. \end{array}$$

*Thus*,

$$\begin{array}{c} \Delta^{2}a_{0}=\frac{1}{6};\;\Delta^{1}a_{1}=-\frac{1}{3};\;\Delta^{0}a_{2}=\frac{1}{6}. \end{array}$$

*Now, using* (5) *and from bottom to top, we can easily derive the following triangle*:

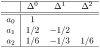


*From this triangle, the pmf’s of X*^(*k*)^, *k* = 0, 1, *are also found, again from* (8):

$$\begin{array}{c} f_{0}=(1);\;f_{1}=(\frac{1}{2},\frac{1}{2}). \end{array}$$

*We now wish to find a feasible value for a*_3_ = *x*. *In order to preserve the condition of cgs, the entries in the additional row must maintain the sign of each column*:

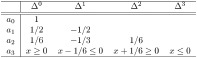

*To conclude*, *x* = 0 *leads to* $f_{3}=(0,\frac{1}{2},\frac{1}{2},0)$, *which is a uniform distribution on* {1, 2}.

For any unknown cgs {1, *a*_1_, …, *a*_*n*_}, the squares of a generic triangle table are easily found as a linear function of *a*_*i*_, by using (6) and (8). Moreover, it is easy to obtain cases where a cgs can be enlarged with a unique feasible additional number, or within a rank of values, or it is impossible, as the last row gives an inequalities system, which can have just one solution, or no solution or an infinite number of solutions.

The nature of the *k*–connection among distributions is illustrated by the following set of results. The proof of each one reduces to simple checking. The parameter notation is shown in the Introduction section.

**Proposition 3** *Let p be a real number within the real interval* [0, 1]. *Then, the sequence* {*a*_*n*_ = *p*^*n*^: *n* ≥ 0} *is a cgs and the chain of distributions generated is the set of classical binomial distributions* {*Bin*(*n*, *p*): *n* = 0, 1, 2, …}.

*Proof*. The proof is immediate from (8).

**Proposition 4** *Given M* ≥ *R* > 0 *integers, the sequence*

$$\begin{array}{c} a_{n}=\{\begin{array}{cc} \frac{R!(M-n)!}{(R-n)!M!}, & n=0,\ldots ,R;\\ 0, & n=R+1,\ldots ,M, \end{array} \end{array}$$

*is a cgs and the chain of distributions generated is the set of hypergeometric distributions* {H(*M*, *N*, *n*): *n* = 0, 1, 2, …, *M*}.

*Proof*. The proof is immediate from (8).

The latter result has an interesting meaning, namely that in sampling without replacement, higher values of *n* lead to null probabilities for some extreme values of *X*^(*n*)^, meaning that its support set is not actually the integer interval [0, *n*], but a subset within it.

**Proposition 5** *Given W* > 0, *R* > 0, *c* > 0 *integers, then the sequence*

$$\begin{array}{c} a_{n}=\frac{{\left(\frac{W}{c}\right)}_{n}}{{\left(\frac{W+R}{c}\right)}_{n}}, \end{array}$$

*for n* ≥ 1 *and a*_0_ = 1 *is a cgs and the chain generated is the set of Pólya distributions* {*P*(*W*, *R*, *c*, *n*): *n* = 0, 1, 2, …}.

*Proof*. The proof is immediate from (8).

**Proposition 6** *The sequence a*_*n*_ = (*n* + 1)^−1^, *for n* ≥ 0 *is a cgs and the chain of distributions generated is the family of discrete uniform distributions in the integer intervals* [0, *n*].

*Proof*. The proof is immediate from (8).

From the previous results, it seems that the *k*–connection of finite distributions is an essential relationship, which is present in a natural way, although largely unnoticed. Accordingly, it seems credible that certain apparently unrelated distributions may actually present a similar relationship, for example, Poisson–Binomial distributions with no common individual probabilities of success. On the other hand, any more or less arbitrary finite distribution can be connected to a chain.

## Estimation

Consider the following scenario. In a given country or city, the presence of infectious disease is noticed, and planners wish to model the number of contagious persons in a classroom, waiting room or similar. An initial approach to this task might be to create a binomial model, whereby the parameter to be estimated would be the proportion of contagious persons, *p* within the total population in the environment. This parameter could be estimated from samples of rooms containing any number of persons. If any model other than a binomial one were considered, the existence of different-sized rooms (i.e. capacities) could make it difficult or even impossible to conduct a joint estimation.

On the one hand, the possibility of making joint use of different room capacities is helpful, as the number of persons within each room is another random variable in itself. But on the other hand, the binomial model requires the (uncomfortable) condition of independence among the numbers present in each room.

Given these circumstances, a helpful, relaxed condition could be to consider that the number of contagious persons in a room of any size is *k*−connected random variables. The implications of this assumption are only the regularity conditions given by the proportionality of the factorial moments for each room size. The advantage of this assumption is that it reduces the problem of estimation to that of finding a cgs, {*a*_*n*_: *n* = 0, …, *M*}, and making joint use of all data, with no constraints on room sizes.

Consider the following notation. There are *N* rooms; inside each room *k*_*j*_ people are meeting, where *j* = 1, …, *N* and each *k*_*j*_ ∈ {1, …, *M*}, and where *M* is the highest number of persons observed in a room. The number of persons infected after each meeting is given by $X_{j}^{\left(k_{j}\right)},\;j=1,\ldots ,N.$ Then, we denote by

$$\begin{array}{c} d(k,i)=\#\{j:k_{j}=k\wedge X_{j}^{\left(k_{j}\right)}=i,\;j=1,\ldots ,N\}, \end{array}$$

the number of cases where *X*^(*k*)^ = *i*, that is, the number of rooms with *k* persons attending and where *i* of them are infected after their meeting. We also denote

$$\begin{array}{c} d_{k}=\sum_{i=0}^{k}d(k,i), \end{array}$$

that is, the number of rooms with *k* persons present. Then, $N=\sum_{i=0}^{k}d_{k}$.

The problem to be addressed is then to estimate

$$\begin{array}{c} f_{k}=(f_{k,0},\ldots ,f_{k,k}), \end{array}$$

where

$$\begin{array}{c} f_{k,i}=\;\text{Pr}\;(X^{\left(k\right)}=i), \end{array}$$

If no assumptions were made for the models, there would be *M*(*M* − 1)/2 values to be estimated, {**f**_*k*_: *k* = 0, …, *M*}. But if we assume that those **f**_*k*_ are *k*–connected pmf’s, the problem reduces to that of estimating the *M* unknown values ot their cgs, {*a*_*k*_: *k* = 0, …, *M*}, where *a*_0_ = 1.

To do so, estimates ${\hat{f}}_{k}$ for **f**_*k*_ can be found by solving the following program:

$$\begin{array}{cc} \text{min} & \sum \|d(k,i)-d_{k}\cdot f_{k,i}\|\\ s.t.: & \;f_{k,i}\geq 0,\;i=0,\ldots ,k,\;k=1,\ldots ,N \end{array}$$ (9)

In this case, the best results are obtained by the quadratic norm ‖*x*‖ = *x*^2^.

Then, by using (6) and (8), each ${\hat{f}}_{k,i}$ can be written as a linear function of {*a*_*k*_: *k* = 0, …, *M*}. These are well–known, and no comments are needed in this paper about convergence ${\left\|d(k,i)-d_{k}\cdot {\hat{f}}_{k,i}\right\|}_{d_{k}\to \infty }\to 0$ (from the law of large numbers) or the chi-square goodness–of–fit test for each pmf, **f**_*k*_.

The following example illustrates how even with a sparse dataset, estimation is feasible.

**Example 3** *Assume an infectious disease outbreak and the known existence of meetings at which some of those present have been infected. At each meeting, there are three, four or five attendees. Consider eight samples, as shown in* Table 1:

**Table 1:** Number of attendees and persons infected at each of the 8 samples per row.

Attendees	Infected
3	0,1,1,2,2,2,3,3
4	1,2,2,3,3,3,4,4
5	1,2,3,3,4,4,4,5

*Here, the values* $X_{j}^{\left(k_{j}\right)}$ *(Infected) from rooms with the same number of attendees, k*_*j*_ = 3, 4, 5 *(Attendees) are shown in each row. For rooms with three attendees, only one meeting concluded with no persons infected; two meetings concluded with one infected, three with two infected and two with three infected*.

*These data can also be presented as follows*:

$$\begin{array}{ccc} d(3,0) & = & 1,\;d(3,1)=2,\;d(3,2)=3,\;d(3,3)=2\\ d(4,0) & = & 0,\;d(4,1)=1,\;d(4,2)=2,\;d(4,3)=3,\;d(4,4)=2,\\ d(5,0) & = & 0\;d(5,1)=1,\;d(5,2)=1,\\ d(5,3) & = & 2,\;d(5,4)=3\;d(5,5)=1. \end{array}$$

*Then, the unknown pmf’s can be written as*

$$\begin{array}{c} f_{3}=[-a_{3}+3a_{2}-3a_{1}+1,3(a_{3}-2a_{2}+a_{1}),3(a_{2}-a_{3}),a_{3}], \end{array}$$

$$\begin{array}{c} f_{4}=\left[a_{4}-4a_{3}+6a_{2}-4a_{1}+1,4(-a_{4}+3a_{3}-3a_{2}+a_{1}),6(a_{4}-2a_{3}+a_{2}),4(a_{4}-a_{3}),a_{4}\right] \end{array}$$

$$\begin{array}{c} f_{5}=[-a_{5}+5a_{4}-10a_{3}+10a_{2}-5a_{1}+1,\ldots ,5(a_{5}-a_{4}),a_{5}]. \end{array}$$

*The programs were solved using Wolfram Mathematica^©^. The results obtained (rounded to 2 decimals) are shown in* Table 2. *Notice that each respective cgs is found on the main diagonal of the corresponding table*.

**Table 2:** Estimated probability mass functions.

	Pr(0)	Pr(1)	Pr(2)	Pr(3)	Pr(4)	Pr(5)
**f**_0_	1	0	0	0	0	0
**f**_1_	0.36	0.64	0	0	0	0
**f**_2_	0.15	0.42	0.43	0	0	0
**f**_3_	0.07	0.23	0.40	0.30	0	0
**f**_4_	0.04	0.15	0.23	0.38	0.20	0
**f**_5_	0.01	0.13	0.13	0.25	0.35	0.13

*The estimate for the connecting function of* **f**_5_ *is given by*

$$\begin{array}{c} C(z)=-0.130124+1.00027z-2.94753z^{2}+4.27716z^{3}-3.18756z^{4}+z^{5}. \end{array}$$

*Its three real roots are*, *z*_1_ = 0.358869, *z*_2_ = 0.489898, *z*_3_ = 0.902781, *and so the estimate of* **f**_5_ *is a Poisson–Binomial pmf*.

## A simulation experiment

Suppose that, in a contagion situation within several rooms, as in the Estimation section, the probability distributions of *X*^(*k*)^ (number of infected persons after a meeting with *k* attendees) are *k*–connected hypergeometric, meaning that *X*^(*k*)^ ∼ H(*M*, *N*, *k*), where *M* and *N* are fixed values for all values of *k* considered, *M* ≥ *k*, and *k* is the number of attendees in each room.

A simulation study was conducted to evaluate the estimation errors, considering three hypergeometric chains H(100, 20, *n*), H(100, 50, *n*) and H(100, 80, *n*). For each chain, only the data from *n* = 5, 10, 20 were simulated.

Two scenarios are considered:

- Scenario 1: Sample sizes were 35, 35, 30 for the respective values of *n* = 5, 10, 20. This first scenario is an almost egalitarian one, while the second would be cheaper (in the example given in the above section about meetings under epidemic situation).
- Scenario 2: Sample sizes were 50, 25, 25 for the respective values of *n* = 5, 10, 20.

1000 simulations of each case were performed and the results obtained are shown in Tables 3 to 8. In each table, the exact values for the cgs (*a*_*i*_) and the last pmf, Pr(*X*^(20)^ = *i*), are shown beside the respective mean squared error (mse) for the estimations. In Tables 3 and 4, the simulated data correspond to H(100, 20, *n*)simulations; in Tables 5 and 6, data belong to H(100, 50, *n*); and in Tables 7 and 8 the data correspond to H(100, 80, *n*) estimations, where each pair of tables corresponds to Scenario 1 and 2, respectively.

**Table 3:** Scenario 1. *H*(100, 20, *n*) where *n* = 5, 10, 20.

*i*	*a*_*i*_	mean *a*_*i*_	mse(*a*_*i*_)	Pr(*i*)	mean Pr(*i*)	mse (Pr(*i*))
0	1	1	0	0.00659594	0.0262467	0.000664715
1	0.2	0.271854	0.00613594	0.0432521	0.0370613	0.000681201
2	0.0383838	0.105385	0.00522223	0.125919	0.101032	0.00361373
3	0.00705009	0.057036	0.00288968	0.215862	0.183343	0.00635286
4	0.00123558	0.0372934	0.00150628	0.243688	0.206126	0.0074745
5	0.000205931	0.026535	0.000809113	0.191951	0.150218	0.00617363
6	0.0000325153	0.0197261	0.000458071	0.109063	0.0702231	0.00318446
7	4.84 × 10^−6^	0.0151093	0.000273676	0.0455786	0.0320194	0.000579272
8	6.77 × 10^−7^	0.0118519	0.000171737	0.0141595	0.0204036	0.00016339
9	8.83 × 10^−8^	0.00948517	0.000112578	0.00328337	0.0185264	0.000306308
10	1.07 × 10^−8^	0.00772356	0.000076744	0.000567554	0.0203981	0.000457889
11	1.186 × 10^−9^	0.00638557	0.0000542178	0.0000726701	0.0196984	0.00044445
12	1.20 × 10^−10^	0.00535212	0.0000395962	6.81 × 10^−6^	0.0203859	0.000467378
13	1.10 × 10^−11^	0.00454284	0.000029837	4.59 × 10^−7^	0.0247557	0.000702795
14	8.77 × 10^−13^	0.00390197	0.0000231607	2.17 × 10^−8^	0.0169341	0.000344913
15	6.12 × 10^−14^	0.00338973	0.0000184911	2.17 × 10^−8^	0.0142907	0.000245178
16	3.60 × 10^−15^	0.002977	0.0000151579	1.43 × 10^−11^	0.0140222	0.000237945
17	1.71 × 10^−16^	0.00264194	0.0000127329	1.75 × 10^−13^	0.0113501	0.00015821
18	6.20 × 10^−18^	0.00236787	0.0000109365	1.12 × 10^−15^	0.00725644	0.0000672437
19	1.51 × 10^−19^	0.00214196	9.58 × 10^−6^	2.98 × 10^−18^	0.00375453	0.0000197057
20	1.86 × 10^−21^	0.00195423	8.54 × 10^−6^	0.125919	0.00195423	8.54 × 10^−6^
Total	–	–	0.000828097	1	–	0.00149539

**Table 4:** Scenario 2. *H*(100, 20, *n*) where *n* = 5, 10, 20.

*i*	*a*_*i*_	mean *a*_*i*_	mse(*a*_*i*_)	Pr(*i*)	mean Pr(*i*)	mse (Pr(*i*))
0	1	1	0	0.00659594	0.029304	0.00105527
1	0.2	0.262825	0.00483402	0.0432521	0.0412223	0.000859336
2	0.0383838	0.0984558	0.00425147	0.125919	0.103989	0.00378119
3	0.00705009	0.0522257	0.00238994	0.215862	0.187097	0.00660327
4	0.00123558	0.033995	0.00126294	0.243688	0.209884	0.00783769
5	0.000205931	0.0242883	0.000690615	0.191951	0.14748	0.00679407
6	0.0000325153	0.0182135	0.000400042	0.109063	0.0690276	0.00357991
7	4.843 × 10^−6^	0.0141107	0.000245375	0.0455786	0.0327003	0.000690876
8	6.7693 × 10^−7^	0.0112135	0.000158236	0.0141595	0.0206662	0.00017422
9	8.8293 × 10^−8^	0.00909877	0.000106449	0.00328337	0.0184538	0.000307339
10	1.0673 × 10^−8^	0.00751255	0.0000742072	0.000567554	0.0194673	0.000420477
11	1.186 × 10^−9^	0.00629532	0.0000533225	0.0000726701	0.0184263	0.000389932
12	1.1998 × 10^−10^	0.00534345	0.000039333	6.813 × 10^−6^	0.0187196	0.000397633
13	1.0903 × 10^−11^	0.00458753	0.0000296908	4.5943 × 10^−7^	0.022139	0.000557509
14	8.772 × 10^−13^	0.00397973	0.0000228797	2.1732 × 10^−8^	0.014776	0.000263712
15	6.120 × 10^−14^	0.00348604	0.0000179644	2.173 × 10^−8^	0.0121336	0.000177651
16	3.600 × 10^−15^	0.00308158	0.0000143493	1.429 × 10^−11^	0.011716	0.000166751
17	1.714 × 10^−16^	0.00274768	0.0000116442	1.747 × 10^−13^	0.00991182	0.000121937
18	6.196 × 10^−18^	0.00247003	9.588 × 10^−6^	1.120 × 10^−15^	0.00692317	0.000062485
19	1.511 × 10^−19^	0.00223751	8.002 × 10^−6^	2.985 × 10^−18^	0.00392164	0.00002222
20	1.866 × 10^−21^	0.00204143	6.762 × 10^−6^	0.125919	0.00204143	6.762 × 10^−6^
Total	–	–	0.000731341	1	–	0.00163192

**Table 5:** Scenario 1. *H*(100, 50, *n*) where *n* = 5, 10, 20.

*i*	*a*_*i*_	mean *a*_*i*_	mse(*a*_*i*_)	Pr(*i*)	mean Pr(*i*)	mse(Pr(*i*))
0	1	1	0	8.793 × 10^−8^	0.019301	0.000423816
1	0.5	0.478273	0.000714287	2.836 × 10^−6^	0.0187427	0.000399058
2	0.247475	0.247407	0.000209011	0.0000412617	0.0201606	0.000469202
3	0.121212	0.132727	0.000310055	0.000360102	0.0165181	0.000325567
4	0.0587316	0.0740286	0.000374546	0.0021156	0.0204372	0.000423312
5	0.0281422	0.0434128	0.000337277	0.0088976	0.0219637	0.000288987
6	0.0133305	0.0270581	0.000262747	0.027805	0.0268727	0.000308576
7	0.00623983	0.0180066	0.000190918	0.0661308	0.0482384	0.00132162
8	0.00288508	0.0127473	0.000134561	0.121602	0.0943245	0.00334131
9	0.0013171	0.00950666	0.0000940776	0.174609	0.146117	0.00516198
10	0.00059342	0.00738351	0.0000661493	0.196871	0.169261	0.00553581
11	0.000263742	0.00591258	0.0000471884	0.174609	0.148333	0.00503247
12	0.000115572	0.00484627	0.0000343374	0.121602	0.101236	0.00333282
13	0.0000499063	0.00404665	0.0000255647	0.0661308	0.0591852	0.00123585
14	0.0000212245	0.0034322	0.0000194983	0.027805	0.0310876	0.000459348
15	8.885 × 10^−6^	0.00295157	0.0000152344	0.027805	0.0196668	0.000270624
16	3.658 × 10^−6^	0.0025704	0.0000121821	0.0021156	0.0157637	0.000277218
17	1.481 × 10^−6^	0.00226456	9.955 × 10^−6^	0.000360102	0.0111275	0.000157651
18	5.887 × 10^−7^	0.00201651	8.298 × 10^−6^	0.0000412617	0.00665356	0.00006173
19	2.297 × 10^−7^	0.00181325	7.04168 × 10^−6^	2.836 × 10^−6^	0.00336493	0.0000180435
20	8.793 × 10^−8^	0.001645	6.073 × 10^−6^	0.0000412617	0.001645	6.073 × 10^−6^
Total	–	–	0.00014395	1	–	0.00137386

**Table 6:** Scenario 2. *H*(100, 50, *n*) where *n* = 5, 10, 20.

*i*	*a*_*i*_	mean *a*_*i*_	mse(*a*_*i*_)	Pr(*i*)	mean Pr(*i*)	mse (Pr(*i*))
0	1	1	0	8.793 × 10^−8^	0.0165672	0.000317122
1	0.5	0.483319	0.000640753	2.836 × 10^−6^	0.0163346	0.000307585
2	0.247475	0.250621	0.000347012	0.0000412617	0.0177904	0.000375308
3	0.121212	0.134502	0.000427707	0.000360102	0.0156997	0.000305505
4	0.0587316	0.0749533	0.000438192	0.0021156	0.0189751	0.000382738
5	0.0281422	0.0438641	0.000366137	0.0088976	0.0211285	0.00028278
6	0.0133305	0.0272526	0.000274392	0.027805	0.0261491	0.000412183
7	0.00623983	0.0180651	0.000195002	0.0661308	0.0490477	0.00153268
8	0.00288508	0.0127371	0.000135618	0.121602	0.0945931	0.00353275
9	0.0013171	0.00946548	0.0000940812	0.174609	0.146485	0.00577509
10	0.00059342	0.00733267	0.0000659103	0.196871	0.17217	0.00643016
11	0.000263742	0.005864	0.0000470087	0.174609	0.152539	0.00580381
12	0.000115572	0.00480628	0.0000343042	0.121602	0.101577	0.00366574
13	0.0000499063	0.00401805	0.00002568	0.0661308	0.0591698	0.00135839
14	0.0000212245	0.00341564	0.0000197367	0.027805	0.0324712	0.000570899
15	8.885 × 10^−6^	0.00294638	0.0000155666	0.027805	0.0210031	0.000357689
16	3.658 × 10^−6^	0.00257521	0.0000125844	0.0021156	0.0160033	0.000302161
17	1.481 × 10^−6^	0.00227773	0.0000104111	0.000360102	0.0109458	0.000160198
18	5.887 × 10^−7^	0.00203636	8.799 × 10^−6^	0.0000412617	0.0063863	0.0000598713
19	2.297 × 10^−7^	0.00183821	7.583 × 10^−6^	2.836 × 10^−6^	0.00329085	0.0000191426
20	8.793 × 10^−8^	0.00167366	6.654 × 10^−6^	0.0000412617	0.00167366	6.654 × 10^−6^
Total	–	–	0.000158657	1	–	0.00152183

**Table 7:** Scenario 1. *H*(100, 80, *n*) where *n* = 5, 10, 20.

*i*	*a*_*i*_	mean *a*_*i*_	mse(*a*_*i*_)	Pr(*i*)	mean Pr(*i*)	mse (Pr(*i*))
0	1	1	0	1.866 × 10^−21^	0.00793569	0.000101726
1	0.8	0.749504	0.00388344	2.985 × 10^−18^	0.00773466	0.0000969905
2	0.638384	0.583925	0.004553	1.120 × 10^−15^	0.00766626	0.0000954767
3	0.508101	0.457955	0.0039429	1.747 × 10^−13^	0.0071716	0.0000843803
4	0.403338	0.359081	0.00315898	1.429 × 10^−11^	0.00734159	0.0000887814
5	0.319309	0.28076	0.00246952	6.954 × 10^−10^	0.00752825	0.0000909079
6	0.252086	0.218592	0.00191599	2.173 × 10^−8^	0.00683131	0.0000760903
7	0.198451	0.169297	0.00148371	4.594 × 10^−7^	0.00723933	0.0000847369
8	0.155773	0.130317	0.00114848	6.813 × 10^−6^	0.00736504	0.000086718
9	0.12191	0.0996113	0.000888455	0.0000726701	0.0077133	0.0000942853
10	0.0951163	0.0755386	0.000686301	0.000567554	0.00845152	0.000108698
11	0.0739793	0.0567715	0.000528731	0.00328337	0.00978918	0.000127513
12	0.0573548	0.0422359	0.000405682	0.0141595	0.015911	0.000270103
13	0.0443196	0.0310629	0.000309534	0.0455786	0.0399379	0.00110113
14	0.0341312	0.0225512	0.00023451	0.109063	0.0975736	0.00319351
15	0.0261937	0.0161363	0.000176219	0.109063	0.176439	0.00555138
16	0.0200305	0.0113651	0.000131311	0.243688	0.236614	0.00582794
17	0.0152613	0.00787584	0.0000972341	0.215862	0.203737	0.006051
18	0.0115839	0.00538072	0.0000720633	0.125919	0.111695	0.00346575
19	0.00875855	0.00365218	0.0000544142	0.0432521	0.0228133	0.00118291
20	0.00659594	0.00251152	0.0000434223	1.1201 × 10^−15^	0.00251152	0.0000434223
Total	–	–	0.00130919	1	–	0.00132493

**Table 8:** Scenario 2. *H*(100, 80, *n*) where *n* = 5, 10, 20.

*i*	*a*_*i*_	mean *a*_*i*_	mse(*a*_*i*_)	Pr(*i*)	mean Pr(*i*)	mse (Pr(*i*))
0	1	1	0	1.86 × 10^−21^	0.00490265	0.0000462427
1	0.8	0.768486	0.00184085	2.98 × 10^−18^	0.00466926	0.0000432453
2	0.638384	0.605351	0.00224843	1.12 × 10^−15^	0.00463977	0.0000423858
3	0.508101	0.478937	0.00205368	1.75 × 10^−13^	0.00429676	0.0000362838
4	0.403338	0.378836	0.00175865	1.43 × 10^−11^	0.00457045	0.0000403688
5	0.319309	0.299035	0.00148432	6.95 × 10^−10^	0.00496864	0.0000473832
6	0.252086	0.235318	0.00124989	2.17 × 10^−8^	0.00465226	0.0000413063
7	0.198451	0.184485	0.00105175	4.59 × 10^−7^	0.00540529	0.0000558015
8	0.155773	0.144018	0.000883612	6.81 × 10^−6^	0.00598145	0.0000673022
9	0.12191	0.111902	0.000740501	0.0000726701	0.00686121	0.0000876842
10	0.0951163	0.086507	0.000618815	0.000567554	0.00807862	0.000120217
11	0.0739793	0.0665102	0.000515782	0.00328337	0.0097998	0.000164977
12	0.0573548	0.0508382	0.000429077	0.0141595	0.0157663	0.000342529
13	0.0443196	0.0386198	0.000356633	0.0455786	0.0368223	0.00124593
14	0.0341312	0.0291494	0.000296588	0.109063	0.0924901	0.00393681
15	0.0261937	0.0218575	0.000247277	0.109063	0.1793	0.00624285
16	0.0200305	0.0162861	0.000207259	0.243688	0.236579	0.00719695
17	0.0152613	0.0120681	0.000175336	0.215862	0.209047	0.00626063
18	0.0115839	0.00891106	0.000150583	0.125919	0.122607	0.00391803
19	0.00875855	0.00658267	0.000132389	0.0432521	0.0336619	0.00134578
20	0.00659594	0.00489957	0.000120517	1.12 × 10^−15^	0.00489957	0.000120517
Total	–	–	0.000828097	1	–	0.00149539

## Conclusions

In the epidemiology of communicable diseases, it is essential to analyse and control the spread of infection, which can provoke severe problems not only for public health but in many other areas (for example, a major outbreak may force schools and universities to close). In this respect, statistical procedures such as contagious statistical distributions can be a useful means of studying and controlling the situation. In this paper, we describe the development of procedures directly linked to the modelling of contagion in a closed environment through *k*–connected chains of distributions.

A chain of *k*–connected distributions contains a single probability distribution for each integer interval [0, *n*] as its support set, where *n* = 0, …, *N*, and where *N* could be infinity. These distributions are closely related, as verified by various well–known models for sampling within a given population and for other families of finite distributions.

Conversely, any probability distribution with a support set in the integer interval [0, *N*] belongs to a chain, which could be enlarged with other distributions with support sets [0, *N* + 1], [0, *N* + 2], …

A major application of this result is as a means of estimating the probabilities of a set of distributions from sparse data, as we show in an example. This example also illustrates how the approach described can be used to obtain a contagious model which contains the Pólya distribution as a particular case. When the hypothesis of *k*–connection among a set of finite distributions is accepted, the pmf of each one can be estimated with data from some of them. Therefore, the *k*–connection might be considered not only a generalisation of the relationship among sampling distributions from a given population, in which different sample sizes can be jointly used to estimate the common probability of success, *p*, but also a generalisation of the Pólya contagious model, as both can be obtained as particular cases of *k*–connected chains.
